# Fine-Tuning DeepSeek-OCR‑2 for Molecular Structure Recognition

**DOI:** 10.1021/acsomega.6c04159

**Published:** 2026-09-14

**Authors:** Haocheng Tang, Xingyu Dang, Junmei Wang

**Affiliations:** † School of Pharmacy, 6614 University of Pittsburgh, Pittsburgh, Pennsylvania 15261, United States; ‡ Department of Computer Science, 6740 Princeton University, Princeton, New Jersey 08540, United States

## Abstract

Optical chemical structure recognition (OCSR) is critical for converting 2D molecular diagrams from printed literature into machine-readable formats. While vision-language models have shown promise in end-to-end OCR tasks, their direct application to OCSR remains challenging, and direct full-parameter supervised fine-tuning often fails. In this work, we adapt DeepSeek-OCR-2 for molecular optical recognition by formulating the task as an image-conditioned SMILES generation. To overcome training instabilities, we propose a two-stage progressive supervised fine-tuning strategy: starting with parameter-efficient LoRA and transitioning to selective full-parameter fine-tuning with split learning rates. We train our model on a large-scale corpus combining synthetic renderings from PubChem and realistic patent images from USPTO-MOL to improve the coverage and robustness. Our fine-tuned model, MolSeek-OCR, demonstrates competitive capabilities, achieving exact-match accuracies comparable to those of the best-performing image-to-sequence model. However, it remains inferior to state-of-the-art image-to-graph models. Exploratory post-training shows mixed results: ReFT provides small benchmark-dependent gains, whereas GSPO does not yield stable improvement and can collapse under extended optimization.

## Graphical abstract

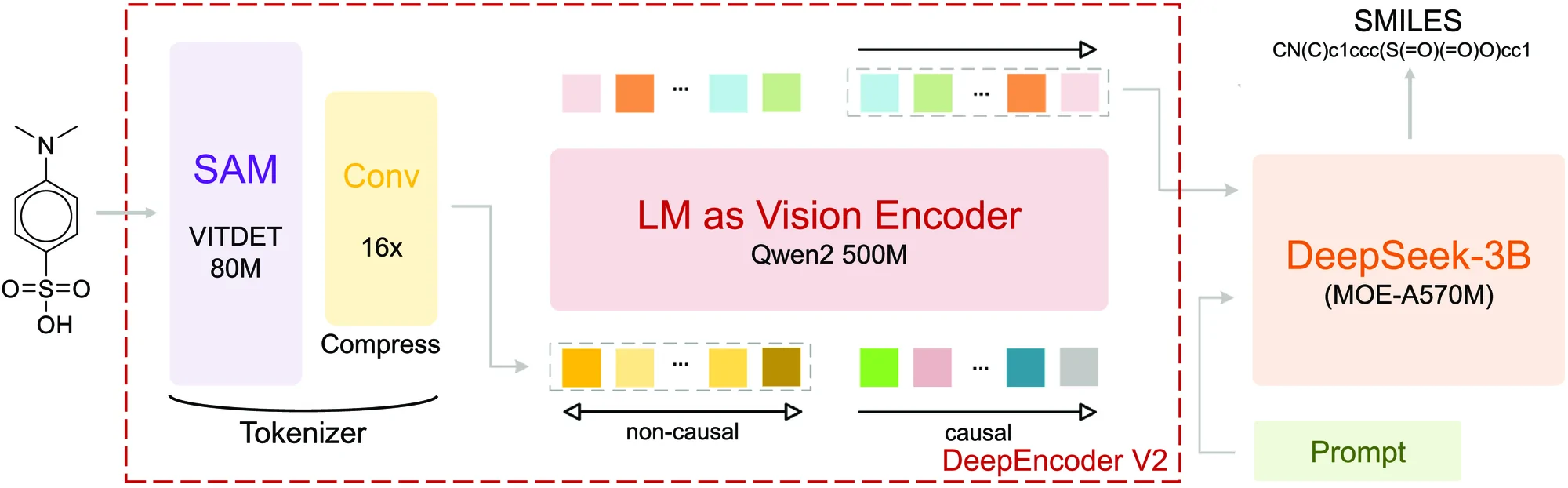

## Introduction

The automated extraction of chemical knowledge relies heavily on the accurate digitalization of complex scientific documents.^1,2^ A critical component of this digitization process is optical chemical structure recognition (OCSR), which aims to convert 2D molecular diagrams from printed literature into machine-readable formats, such as SMILES or molecular graphs. Historically, deep learning models for OCSR have evolved from Transformer-based encoder–decoder architectures that translate image pixels directly into SMILES strings, such as DECIMER,^3^ to more advanced approaches like MolScribe^4^ that explicitly predict atoms, bonds, and their geometric layouts to construct robust 2D graphs.

Concurrently, the rapid development of large language models (LLMs) and vision-language models (VLMs) has transformed how AI interacts with chemical data. To overcome the limitations of text-only representations, multimodal LLMs have incorporated structured molecular inputs to enable joint reasoning over the language and molecular structures. In the broader domain of document understanding, fine-tuning VLMs for OCR has increasingly shifted toward end-to-end, OCR-free architectures, eliminating the reliance on pipeline-based OCR engines. These fine-tuned document VLMs provide the essential text and layout grounding required for multimodal chemistry models to autonomously ingest and reason about unstructured scientific literature.

Building on these parallel advancements in the OCSR and document VLMs, this work adapts DeepSeek-OCR-2 ^1,2^ to molecular optical recognition by formulating the task as image-conditioned SMILES generation. DeepSeek-OCR-2 is a document-oriented VLM designed for the OCR-free recognition of text and layout from images, making it a suitable foundation for testing whether document OCR models can be transferred to molecular diagrams. Because direct full-parameter supervised fine-tuning (SFT) failed, we introduced a two-stage progressive supervised fine-tuning strategy. Our approach begins with parameter-efficient fine-tuning using LoRA to adapt the text generation pathway and the cross-modal alignment interface. Subsequently, we apply progressive full-parameter fine-tuning, freezing the lowest-level visual tokenizer while optimizing higher-level modules by using split learning rates for the visual and language branches.

To ensure robustness across diverse drawing styles and document artifacts, we utilize a mixed training dataset combining large-scale synthetic data from PubChem with realistic patent images from USPTO-MOL.^5–8^ Finally, we benchmark our resulting models against traditional baselines to evaluate their exact-match accuracy across synthetic, realistic, and perturbed datasets. We also conducted exploratory experiments with reinforcement-style post-training methods, such as Group Sequence Policy Optimization (GSPO) and Representation Finetuning (ReFT), to examine whether reward-based optimization could improve performance without sacrificing strict sequence-level fidelity. The results highlight the unique challenges of maintaining strict sequence-level fidelity in VLM-based molecular recognition.

## Related Work

### LLMs for Chemistry

LLMs are increasingly utilized in chemistry for molecular understanding and scientific knowledge grounding. Initial applications relied heavily on prompt engineering, proving that general-purpose models could tackle chemical problems without parameter updates.^9–11^ This success inspired chemistry-specific fine-tuned models—such as ChemLLM,^12^ ChemDFM,^13^ and BatGPT-Chem^14^—which adapt robust pretrained backbones to specialized chemical corpora. However, recognizing the limitations of text-only data for complex structural parsing, recent multimodal models have incorporated structured inputs like SMILES, 2D graphs, and 3D geometries. Frameworks like nach0,^15^ InstructMol,^16^ Chem3DLLM,^17^ and ChemVLM^18^ exemplify this shift, laying the essential foundation for joint visual-linguistic reasoning over molecular structures.

### Molecular Structure Recognition

OCSR is critical for converting 2D molecular diagrams from printed literature into machine-readable formats like SMILES or molecular graphs. While early deep learning models such as DECIMER^3^ utilized Transformer-based encoder–decoder architectures to translate image pixels directly into SMILES strings, subsequent approaches like MolScribe^4^ advanced the field by explicitly predicting atoms, bonds, and their geometric layouts to construct robust 2D graphs. More recently, VLMs have been adapted for OCSR. To overcome the limitations of treating molecular recognition merely as a holistic image-captioning task, cutting-edge frameworks like GTR-VL^19^ introduce a “Visual Chain of Thought” mechanism. This approach mimics human reasoning by incrementally parsing molecular graphs through sequential atom-bond predictions, significantly improving the recognition of complex and hand-drawn structures.

### Fine-Tuning VLMs for OCR Tasks

The automated extraction of chemical knowledge relies heavily on the accurate digitization of complex scientific documents. Consequently, fine-tuning VLMs for Optical Character Recognition has increasingly shifted towards end-to-end, OCR-free architectures. Models like Donut^20^ eliminate the reliance on pipeline-based OCR engines by directly mapping document images to structured text using a vision encoder and text decoder. For scientific literature specifically, Nougat^21^ adapts this paradigm to convert dense academic PDFs—including tables, inline math, and chemical equations—directly into Markdown markup. These fine-tuned document VLMs provide the essential text and layout grounding required for multimodal chemistry models to autonomously ingest and reason over unstructured scientific literature Figure 1.

**Figure 1:**
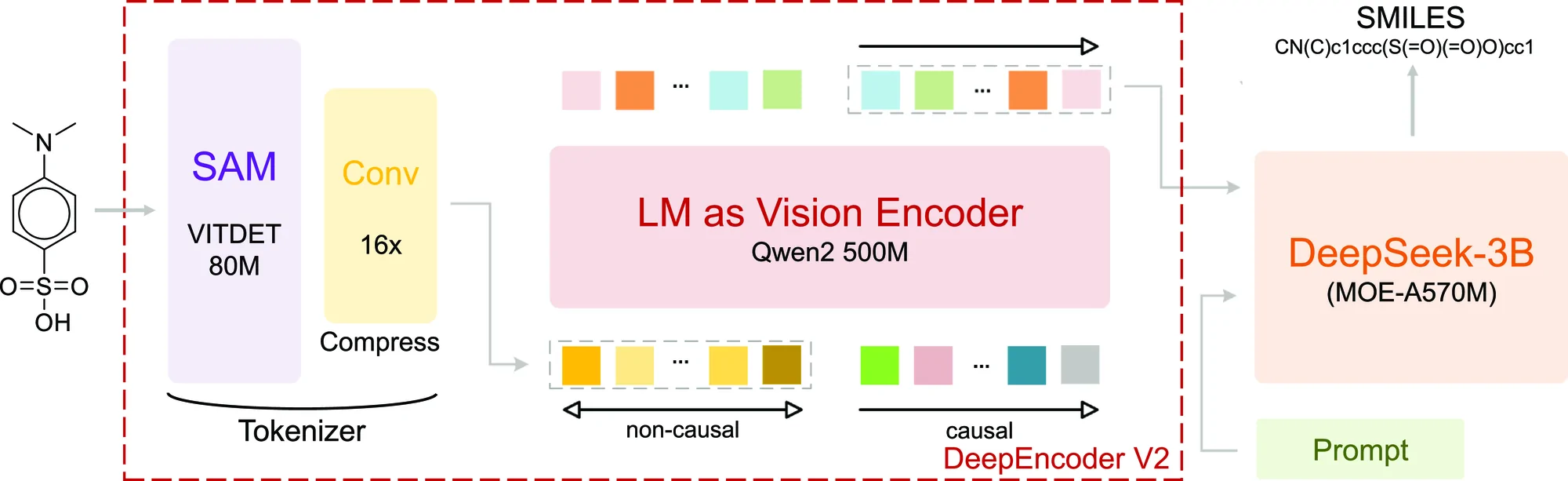
The overall architecture of DeepSeek-OCR-2. In the figure, MoE denotes mixture-of-experts, and A570M indicates approximately 570 million activated parameters.

## Methodology

### Dataset

#### Training

Following the common practice in molecular structure recognition, we combine large-scale synthetic data with realistic patent images to improve both coverage and robustness, following MolScribe. Our training corpus was built from two sources. The first source is PubChem,^7^ which provides a large collection of molecular structures paired with SMILES strings. We used these structures to render molecular depictions on the fly, allowing the model to observe diverse drawing styles during training. In particular, we generated two complementary synthetic domains: a MolScribe-like style with stronger appearance variation and a cleaner ChemDraw-like style with fewer perturbations. This synthetic branch improves the model’s tolerance to style diversity, bond rendering differences, and local annotation variations. The second source is USPTO-MOL,^4^ which contains realistic molecular images extracted from patents together with paired structure annotations. This branch exposes the model to real document artifacts, including imperfect scan quality, nonuniform line thickness, and patent-specific drawing conventions.

The two training stages used the same data sources but different sampling budgets. In the LoRA stage, we sample 64k examples from each of the three subsets, namely, PubChem rendered in the MolScribe-like style, PubChem rendered in the ChemDraw-like style, and realistic USPTO-MOL images, resulting in 192k training instances in total. In the subsequent progressive full fine-tuning stage, we enlarged the corpus to 800k examples, consisting of 300k MolScribe-like rendered molecules from PubChem, 300k ChemDraw-like rendered molecules from PubChem, and 200k realistic molecular images from USPTO-MOL. Across all subsets, the supervision target was the corresponding SMILES sequence.

#### Evaluation

We evaluate the model on both clean and perturbed benchmarks that cover synthetic renderings, patent images, catalog images, and journal figures^5,6^ (Table 1). The clean benchmark suite contains two synthetic sets, namely, ChemDraw and Indigo, and five realistic sets, namely, USPTO, CLEF, Staker, UOB, and ACS. The two synthetic sets measure in-domain recognition under standardized rendering engines, whereas the realistic sets evaluate generalization to authentic molecular depictions collected from patents, catalogs, and journals. In addition, we report robustness on four perturbation sets derived from CLEF, Staker, UOB, and USPTO, where the input images are further corrupted by appearance variations and quality degradation. This evaluation protocol reflects the central challenge of the OCSR: strong performance is required not only on clean synthetic drawings but also on heterogeneous real-world images and visually perturbed inputs.

**Table 1:** Summary of the Benchmarks for Evaluation

Dataset	Source	No. of Images	% of chiral
Indigo	synthetic	5719	20.2%
ChemDraw	synthetic	5719	20.2%
CLEF	patent (US)	992	32.7%
UOB	catalog	5740	0%
USPTO	patent (US)	5719	20.2%
Staker	patent (US)	50,000	17.3%
ACS	journal article	331	19.3%

### Train–Test Overlap and Similarity Audit

Because our training sources follow the same broad data design used by MolScribe, combining PubChem-rendered structures with USPTO-derived patent images, we further quantified possible train–test leakage. We reconstructed the molecules sampled in both supervised stages and deduplicated them by the RDKit standard InChIKey. This analysis produced 458,785 cumulative unique valid training structures. This number is distinct from the LoRA-only 64k × 3 image–text instance count because the two PubChem rendering branches can share molecule identities and because the progressive full-SFT stage uses a larger sampled corpus. Across 159,140 valid benchmark rows, 7852 rows (4.93%) had an exact InChIKey match to the cumulative training corpus (Figure 2A). Exact-match accuracies for MolSeek-OCR-LoRA and MolSeek-OCR after removing these exact InChIKey overlaps are reported in the Supporting Information (Table S11). On most benchmarks, the accuracy differs from the original accuracy by less than 2 percentage points; ACS is the main exception, where removing the relatively high-overlap subset lowers accuracy by about 4 percentage points.

**Figure 2:**
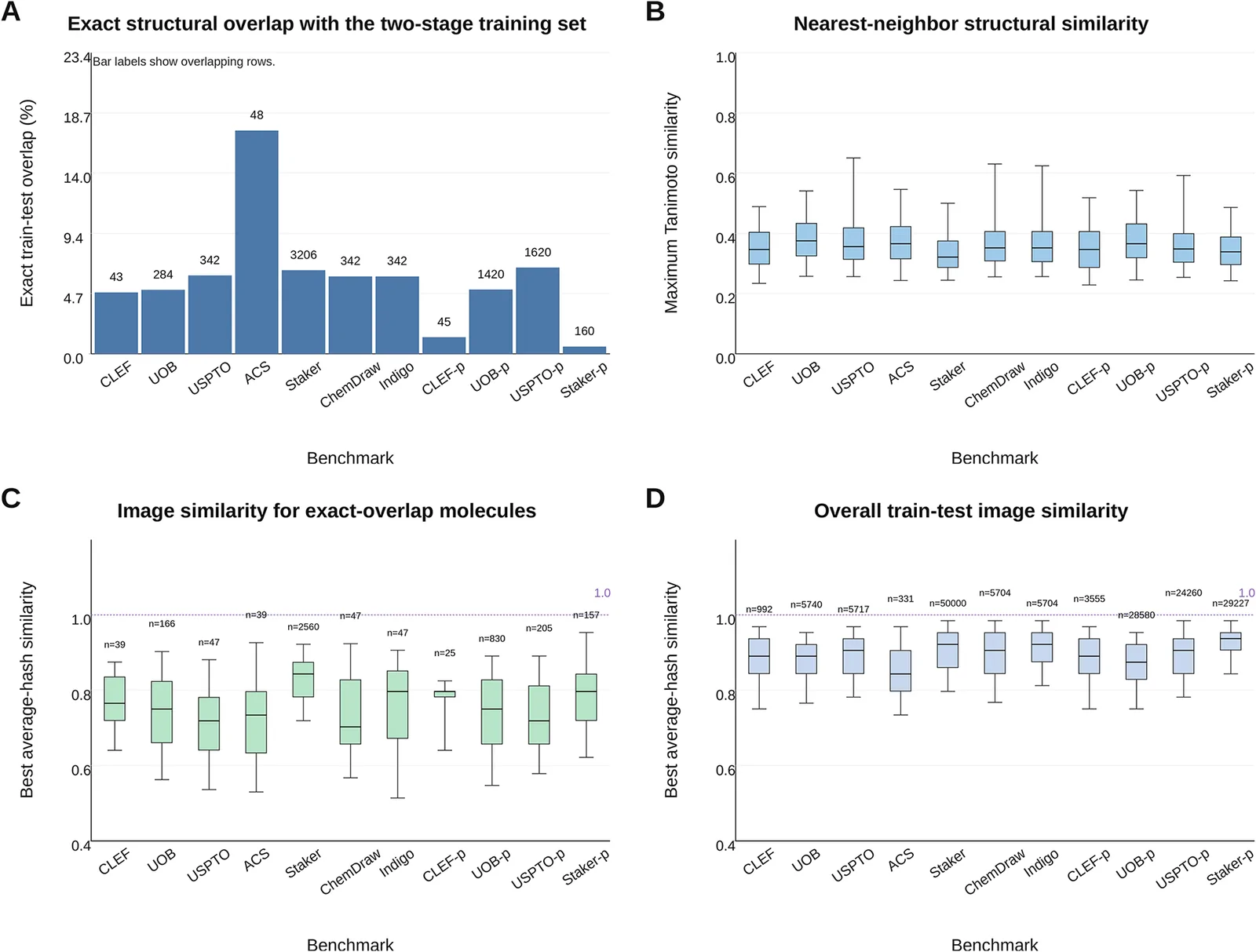
Train–test leakage audit. (A) Exact structural overlap with the cumulative two-stage training set, computed with RDKit standard InChIKey. Bar labels indicate overlapping benchmark rows. (B) Sampled nearest-neighbor Morgan-fingerprint Tanimoto similarity after exact overlaps were removed. (C) Image similarity for exact-overlap molecules, using stored USPTO-MOL training images when available and deterministic re-rendered PubChem images for dynamic-rendering training structures. (D) Overall train–test image similarity, computed as the nearest average-hash similarity from every benchmark image to a sampled training image library.

We also assessed near-neighbor similarity after removing exact structural overlaps. A sampled Morgan-fingerprint Tanimoto analysis showed modest nearest-neighbor similarity across benchmarks, with a median maximum similarity of approximately 0.32–0.38 (Figure 2B). To complement the structure-level audit, we compared image-level similarity using average-hash similarity. The training image library included stored USPTO-MOL images and PubChem molecules deterministically re-rendered with the same RDKit dynamic-rendering logic used during training. The exact-overlap image comparison indicates that identical molecular structures generally do not correspond to identical image instances (Figure 2C). The all-row benchmark image comparison is relatively high because molecular depictions share sparse black-line graphical conventions, even when the underlying structures are different (Figure 2D), especially for InChIKey exact-overlapping molecules. The image-level audit therefore reduces concern about direct image duplication, but does not completely eliminate the possibility that exact structure-level overlap affects a small part of the reported benchmark performance, which is also included in the other benchmark models.

### Training Pipeline

We adapted DeepSeek-OCR-2 to molecular optical recognition by formulating the task as image-conditioned SMILES generation. Given a molecular depiction and a fixed instruction prompt, the model autoregressively predicts the target SMILES sequences. Our training recipe follows a two-stage, progressive supervised fine-tuning strategy. Direct full-parameter SFT failed. In both stages, we preserve the original multi-scale visual pipeline of DeepSeek-OCR-2: each sample is processed with one global view and optional local crops so that both overall molecular topology and local bond-level details can be retained. Supervision is applied only to the generated answer tokens, namely, the target SMILES string, rather than to the prompt or image placeholder tokens.

#### Architecture of DeepSeek-OCR-2

As illustrated in Figure 1, DeepSeek-OCR-2 consists of three main components: a visual tokenizer, an LM-as-vision-encoder module, and an autoregressive decoder, with a compression/projection interface connecting the visual and language streams. The visual tokenizer converts the molecular image into a compact sequence of visual tokens, while the LM-style visual encoder reorganizes and refines these tokens before passing them on to the decoder. This design is well-suited to molecular OCR because it combines fine-grained visual perception with sequential chemical string generation. In particular, the encoder can preserve structural cues such as rings, branches, and bond patterns, whereas the decoder maps the reordered visual representation into a valid SMILES sequence.

#### LoRA SFT

In the first stage, we performed parameter-efficient supervised fine-tuning with LoRA starting from the released DeepSeek-OCR-2 checkpoint. Rather than adapting only the final decoder, we place LoRA modules on the main attention and feed-forward projections, as well as on the visual-language projection layers highlighted by Figure 1. Consequently, this stage adapts not only the text generation pathway but also the alignment interface between the visual encoder and the decoder, which is important for translating molecular drawings into chemical strings (Figure 3A,B).

**Figure 3:**
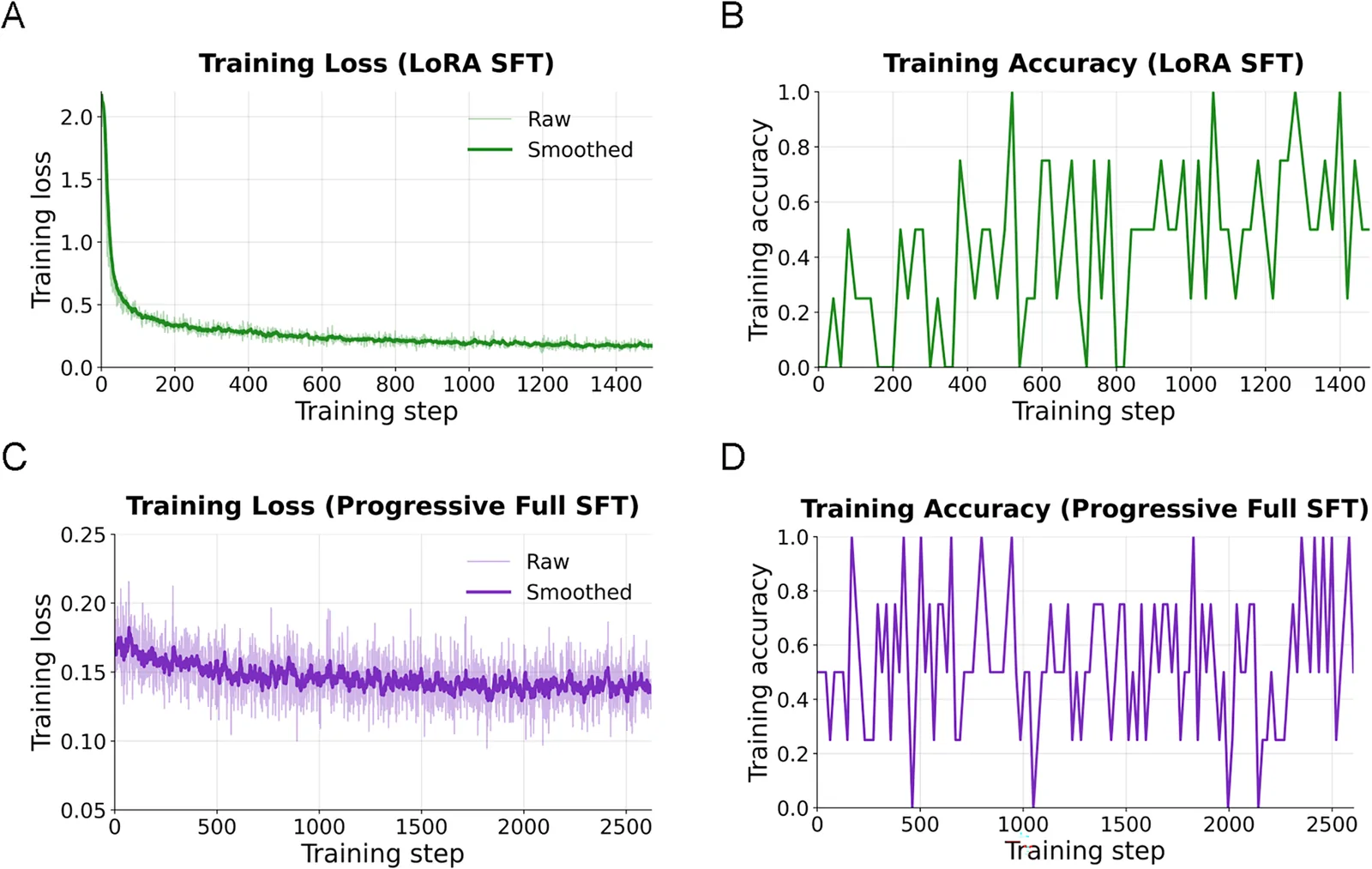
Two-stage fine-tuning. (A) Train loss in stage LoRA SFT. (B) Train accuracy in stage LoRA SFT. (C) Train loss in stage Progressive Full SFT. (D) Train accuracy in stage Progressive Full SFT.

The LoRA stage uses a three-source mixed training set with 64k samples drawn from each source, resulting in 192k training instances in total. Two subsets were constructed from PubChem molecules using on-the-fly rendering. One subset follows a MolScribe-like style with stronger appearance augmentation, while the other follows a cleaner ChemDraw-like style with less augmentation. A third subset is built from realistic molecular images from USPTO-MOL. For synthetically rendered samples, the supervision target is the SMILES string corresponding to the rendered depiction; for realistic samples, the target is the paired SMILES annotation provided with the image. All samples are trained with the same instruction, asking the model to return the SMILES of the input molecule.

#### Progressive Full SFT

In the second stage, we continued training from the LoRA-adapted checkpoint and switched to progressive full-parameter fine-tuning. The training set is expanded to 800k samples, including 300k MolScribe-like rendered molecules from PubChem, 300k ChemDraw-like rendered molecules from the same source, and 200k realistic molecular images from USPTO-MOL. The prompt format and response-only objective remain unchanged.

This stage does not update the entire network uniformly. Instead, we freeze the lowest-level visual tokenizer in Figure 1 together with the input token embedding layer, and continue optimizing the higher-level LM-as-vision-encoder module, the compression/projection interface, and the autoregressive decoder. We further use split learning rates for different branches: a smaller rate for the visual branch and a larger rate for the language-generation branch. This design reflects the intuition that low-level visual perception is already reasonably stable after the first stage, whereas higher-level cross-modal alignment and sequence generation require more task-specific adaptation for molecular OCRs (Figure 3C,D).

## Results

### Performance of SFT Models

As detailed in Table 2, the fine-tuned VLM models demonstrate competitive chemical structure recognition capabilities compared to traditional baselines. The comparison with DECIMER is dataset-dependent: MolSeek-OCR is stronger on several patent, perturbed, and realistic benchmarks, whereas DECIMER remains stronger on others such as Indigo, ChemDraw, and UOB. Thus, MolSeek-OCR should be viewed as broadly competitive with image-to-sequence OCSR, rather than uniformly superior. A simple binomial uncertainty analysis is provided in the Supporting Information (Table S5); it indicates that small differences on smaller benchmarks such as ACS should be interpreted cautiously, whereas the larger gains from MolSeek-OCR over MolSeek-OCR-LoRA on most larger benchmarks exceed the conservative sampling uncertainty range. However, despite these improvements over the zero-shot capabilities of the base model, the VLM-based approaches are still inferior to state-of-the-art image-to-graph models. MolScribe consistently outperforms the SFT VLMs by a significant margin in graph/no-stereo evaluation, suggesting that explicitly predicting geometric node and edge layouts currently retains an advantage over autoregressive text generation for complex structural parsing. MolScribe and GTR-VL^19^ are widely regarded as state-of-the-art methods in this area.

**Table 2:** Benchmark Accuracy (%) on Different Datasets^e^

		Synthetic			Realistic				Perturbed			
Models	Method	Indigo	ChemDraw	CLEF	UOB	USPTO	Staker	ACS	CLEF_*p*_	UOB_*p*_	USPTO_*p*_	Staker_*p*_
Rule-based	MolVec^a^	95.4	87.9	82.8	80.6	88.4	0.8	47.4	43.7	74.5	29.7	5.0
	OSRA^a^	95.0	87.3	84.6	78.5	87.4	0.0	55.3	11.5	68.3	4.0	4.6
Image-to-sequence	Img2Mol^a^,^c^	58.9	46.4	18.3	68.7	26.3	17.0	23.0	21.1	74.9	29.7	51.7
	DECIMER^b^	69.6	86.1	62.7	**88.2**	41.1	40.8	46.5	70.6	**87.3**	46.4	47.9
	SwinOCSR^a^	74.0	79.6	30.0	44.9	27.9	–	27.5	32.2	–	–	–
Image-to-graph	MSE-DUDL^a^	–	–	–	–	–	77.0	–	–	–	–	–
	ChemGrapher^a^	–	–	–	70.6	–	–	–	–	–	–	–
	Image2Graph^a^	–	–	51.7	82.9	55.1	–	–	–	–	–	–
	MolScribe^b^,^d^	**98.1**	**93.9**	**91.7**	88.1	**94.5**	**95.4**	**76.7**	**94.2**	86.9	**93.9**	**87.9**
VLM-based	DeepSeek-OCR-2^b^	0.0	0.0	0.0	0.2	0.0	0.0	0.0	0.0	0.1	0.0	0.0
	MolSeek-OCR-LoRA^b^	68.4	65.1	54.8	64.3	50.6	46.3	24.8	62.0	44.0	56.2	30.0
	MolSeek-OCR^b^	74.3	72.2	63.3	72.6	61.0	50.5	29.9	70.7	49.7	65.6	31.2

a The results are taken from prior reports.

b Results were evaluated in this work for the listed benchmark images.

c Chirality was not predicted.

d Reported by graph/no-stereo accuracy; sequence-generating methods are reported by canonical SMILES exact-match accuracy.

e – means not available.

### Case-Study Error Analysis

Representative examples in the Supporting Information (Table S9 and Figure S2) provide a more chemistry-centered view of these aggregate results. Fine-tuning often corrects malformed or incomplete base-model outputs on patent, catalog, and journal images, including examples with chiral centers, heteroaromatic rings, and perturbed visual styles. The remaining failures are chemically informative: the model can omit salt components, struggle with R-group notation, alter substituent patterns, or preserve the graph-level structure while failing to provide strict stereochemical SMILES serialization. Similar topology or unsaturation errors can also occur on clean synthetic drawings, indicating that not all failures arise from the image noise. These cases support our conclusion that autoregressive SMILES generation can be effective after supervised fine-tuning, but explicit image-to-graph parsing remains more robust for exact structural recovery in difficult or nonstandard depictions.

### Exploratory GSPO and ReFT Post-Training

Beyond progressive SFT, we also explored reinforcement-style post-training with GSPO and data-curation-based refinement with ReFT. In both cases, the starting point was a progressively fine-tuned model. The overall pipeline was to first sample multiple candidate SMILES sequences for each molecular image, then score these candidates with a chemistry-aware reward, and finally use the selected signals to further update the model. The reward was designed to jointly encourage chemical validity, sequence-level correctness, graph-level consistency after removal of chirality-sensitive distinctions, and better handling of stereochemical cases. For evaluation, exact-match accuracy was computed after RDKit SMILES canonicalization, while graph/no-stereo accuracy disabled chirality during canonicalization; the detailed reward definitions and evaluation metrics are reported in the Supporting Information. We also tested a broad range of training settings and reward trade-offs during this stage, aiming to balance exploration with that of stable optimization.

The results of these exploratory experiments were mixed (Table 3). ReFT produced small exact-match gains on several benchmarks; for UOB, UOB-p, USPTO-p, and staker, the improvement exceeded the conservative 95% half-width for a two-model difference, suggesting that part of the ReFT gain was unlikely to be explained by sampling uncertainty alone. The full Supporting Information further shows that ReFT also modestly improved graph/no-stereo accuracy on most benchmarks, although the effect was generally small and not uniform. In contrast, GSPO at global step 200 produced only negligible changes relative to progressive full SFT: all exact-match changes were within the corresponding conservative uncertainty range, and graph/no-stereo changes were also very small, providing no clear evidence of a graph-level improvement. The additional GSPO checkpoint comparison in the Supporting Information further shows no statistically meaningful gain between early checkpoints such as steps 100 and 200, while extended optimization can become unstable and collapse, as reflected by the step-400 result on UOB. Given the substantial additional sampling and training cost, these post-training methods were not adopted in the final training recipe.

**Table 3:** Change in Exact-Match Success Rate after Exploratory Post-Training Relative to Progressive Full SFT

Benchmark	ReFT Δ exact	GSPO Δ exact	95% half-width
CLEF	+0.90	–0.21	4.40
CLEF-p	–0.17	+0.09	2.32
UOB	+2.34	+0.26	1.83
UOB-p	+1.41	+0.15	0.82
USPTO	+0.79	–0.03	1.83
USPTO-p	+1.40	+0.09	0.89
ACS	–0.30	–0.30	7.62
ChemDraw	+0.26	–0.05	1.83
Indigo	+1.57	+0.04	1.83
staker	+1.51	+0.12	0.62
staker-p	+0.73	+0.02	0.81

## Discussion

Through a two-stage progressive fine-tuning strategy, we successfully established a stable transfer path from a document OCR foundation model to molecule-to-SMILES recognition. The resulting model, MolSeek-OCR, demonstrates competitive capabilities, achieving exact-match accuracies that are broadly comparable to those of DECIMER, the best-performing Image-to-Sequence baseline. Despite these advancements, our VLM-based approach remains inferior to state-of-the-art image-to-graph models like MolScribe, indicating that explicitly predicting geometric node and edge layouts is currently superior to autoregressive text generation for structural parsing. Additionally, our exploratory post-training experiments showed that ReFT can provide small benchmark-dependent gains, whereas GSPO did not produce stable improvements and was prone to collapse under extended optimization. Considering the additional training cost, we retained the progressive full-SFT model as our final model.

## Supplementary Material



## Data Availability

All training code, configurations, and evaluation scripts are available at https://github.com/HaCTang/MolSeek-OCR. The reported results correspond to commit 7c1cb69. The released LoRA weight and Progressive Full SFT weight are available on Hugging Face at https://huggingface.co/Haocheng1/MolSeek-OCR/tree/main/LoRA-weight and https://huggingface.co/Haocheng1/MolSeek-OCR/tree/main/Progressive-Full-SFT-weight, respectively. The code is released under the MIT License; datasets, base models, and fine-tuned weights follow the licenses or availability conditions of their original sources.

## Notes

Additional experimental details on SFT and RL (PDF)

## §

Khoury College of Computer Science, Northeastern University, Boston, Massachusetts 02115, United States
