# Supplementary material for: Fine-Tuning DeepSeek-OCR‑2 for Molecular Structure Recognition
Source: ACS Omega. 2026 Sep 14;11(38):57132–8. doi: 10.1021/acsomega.6c04159 (PMC13629064; doi:10.1021/acsomega.6c04159)
Supplement: Supplementary file 1 [file ao6c04159_si_001.pdf]

Fine-tuning DeepSeek-OCR-2 for Molecular Structure Recognition

Haocheng Tang,[*,†,¶] Xingyu Dang,[‡] and Junmei Wang[*,†]

†*School of Pharmacy, University of Pittsburgh, Pittsburgh, PA, 15261, United States*

‡*Department of Computer Science, Princeton University, Princeton, NJ, 08540, United States*

¶*Present address: Khoury College of Computer Science, Northeastern University, Boston, MA, 02115, United States*

E-mail: tang.haoc@northeastern.edu; juw79@pitt.edu

Supplementary Information

Training Details

Tables S1–S4 summarize the final training and post-training settings. Dynamic PubChem entries were rendered online; their image counts therefore denote image–SMILES training instances.

For reproducibility, the exact repository state corresponding to the reported Table 2 results is commit 7c1cb69 (full hash 7c1cb69385b3fd17ec6b2251c85e6ab88b0834e2) of the public repository at `https://github.com/HaCTang/MolSeek-OCR`. The reported MolSeek-OCR-LoRA model was trained with *lora_sft_config.yaml*, merged with *merge_lora_weight.py*, and evaluated from the LoRA checkpoint after merging into the base DeepSeek-OCR-2 model; this released LoRA weight is available at `https://huggingface.co/Haocheng1/MolSeek-OCR/tree/main/LoRA-weight`. The reported MolSeek-OCR model was initialized from the merged LoRA model, trained with *progressive_sft_config.yaml*, and evaluated from the progressive full-SFT checkpoint; this released Progressive Full SFT weight is available at `https://huggingface.co/Haocheng1/MolSeek-OCR/tree/main/Progressive-Full-SFT-weight`. Table 2 evaluation used *evaluation_config.yaml*, while *evaluation_deepseekocr_config.yaml* was used for the base DeepSeek-OCR-2 reference runs. The configurations under *gspo/* and *reft/* correspond to exploratory post-training experiments and are not part of the final paper recipe.

To make the final experimental setup self-contained, Tables S1 and S2 summarize the base checkpoint, prompt-related rendering settings, image size, base size, crop mode, LoRA target modules, frozen modules in progressive full SFT, training-set sizes, random seed, optimization schedule, and hardware/process settings used for the reported supervised models. The code is released under the MIT License. The base DeepSeek-OCR-2 model and released fine-tuned weights follow the DeepSeek-OCR-2 model license. PubChem-derived structures are used under the PubChem terms, USPTO-MOL follows its original release terms, and each benchmark dataset remains subject to the license or access conditions of its original source.

Table S1: LoRA SFT configuration.

Category	Item	Parameter
Model	Initialization	DeepSeek-OCR-2 base checkpoint
Data	PubChem dynamic pool	64k MolScribe-like renders + 64k ChemDraw-like renders
	USPTO-MOL realistic pool	64k loaded realistic images
Sampling	Seed	3407
Rendering	Image size	768
	Base size	1024
	Crop mode	Enabled
	Condensed groups	Disabled
	Molecule augmentation	Enabled for MolScribe-like PubChem renders; disabled for ChemDraw-like renders
LoRA	Rank	64
	Scaling factor	64
	Dropout	0.1
	Target modules	Attention, MLP, projector, and visual projection layers
Optimization	Optimizer	AdamW fused
	Learning rate	2×10^{-4}
	Weight decay	10^{-3}
	Warmup	100 steps
Schedule	Per-device batch size	4
	Gradient accumulation	8
	Training processes	4
	Effective batch size	128
	Training length	3000 updates; approx. 2 passes

LoRA SFT was used as the cold-start adaptation stage. It introduced low-rank adapters into the language, projector, and visual projection modules while keeping the base model largely fixed. This stage established the molecule-image to SMILES instruction-following behavior from a balanced mixture of synthetic-style and realistic molecular images.

Progressive full SFT was then applied after merging the LoRA adapter weights. In this stage, selected full-model parameters were updated with smaller learning rates while low-level visual and embedding components were frozen. The goal was to refine molecule-recognition behavior at larger data scale without destabilizing the pretrained OCR representation.

ReFT was evaluated as an exploratory rejection-sampling fine-tuning stage starting from the progressive full-SFT model. For each prompt, multiple SMILES candidates were generated, scored by chemical reward components, and filtered before a short SFT update on the retained best candidates.

GSPO was evaluated as an exploratory policy-optimization stage using the same molecule-to-SMILES prompt format. It generated multiple responses per prompt with vLLM and optimized the policy with GRPO-style advantages and GSPO clipping, using the same chemically

Table S2: Progressive full SFT configuration.

Category	Item	Parameter
Model	Initialization	Merged LoRA SFT checkpoint at update 3000
Data	PubChem dynamic pool	300k MolScribe-like renders + 300k ChemDraw-like renders
	USPTO-MOL realistic pool	200k loaded realistic images
Sampling	Seed	3407
Rendering	Image size	768
	Base size	1024
	Crop mode	Enabled
	Condensed groups	Disabled
	Molecule augmentation	Enabled for MolScribe-like PubChem renders; disabled for ChemDraw-like renders
Trainable scope	Frozen modules	Visual encoder and token embeddings
	Language-layer freezing	None by layer index
Optimization	Optimizer	AdamW fused
	Base learning rate	1×10^{-5}
	Visual-branch learning rate	5×10^{-6}
	Language-branch learning rate	2×10^{-5}
	Weight decay	0.01
	Warmup	250 steps
	Gradient checkpointing	Enabled
Schedule	Per-device batch size	4
	Gradient accumulation	8
	Training processes	10
	Effective batch size	320
	Training length	2500 updates; approx. 1 pass

motivated reward components as the ReFT exploration but with different weights.

Table S3: ReFT configuration.

Category	Item	Parameter
Model	Initialization	Progressive full-SFT checkpoint at update 2000
Prompt data	PubChem dynamic pool	63,999 MolScribe-like renders + 64k ChemDraw-like renders
	USPTO-MOL realistic pool	32k loaded realistic images; 159,999 total prompts
Sampling	Seed	3407
	Render workers	16
	Render chunk size	64
Input	Image size	768
	Base size	1024
	Crop mode	Enabled
Generation	Candidates per prompt	8
	Temperature	0.3
	Top-p	0.95
	Maximum response length	256 tokens
Filtering	Generated candidates	1,279,992
	Retained samples	121,383
	Retention rate	9.48%
	Retention rule	Keep the best candidate when reward ≥ 0.5
Reward	Validity	0
	Tanimoto	5
	Canonical SMILES	5
	Graph	2
	Chiral	1
	Repetition penalty	2
SFT	Optimizer	AdamW fused
	Base learning rate	5×10^{-6}
	Visual-branch learning rate	2×10^{-6}
	Language-branch learning rate	1×10^{-5}
	Weight decay	0.01
	Warmup	100 steps
	Per-device batch size	4
	Gradient accumulation	8
	Training processes	8
	Effective batch size	256
	Training length	1 epoch over retained samples
	Frozen language layer	0
	Frozen modules	Visual encoder and token embeddings

Table S4: GSPO configuration.

Category	Item	Parameter
Model	Initialization	Progressive full-SFT checkpoint at update 2000
Prompt data	PubChem dynamic pool	12.8k MolScribe-like renders + 12.8k ChemDraw-like renders
	USPTO-MOL realistic pool	6.4k loaded realistic images; 32k total prompts
Sampling	Seed	3407
	Prompt length	3072 tokens
	Response length	128 tokens
	Candidates per prompt	8
Rollout	Temperature	0.3
	Top-p	0.95
	Tensor parallel size	2
	Generated rollout responses	256,000
Reward	Validity	0
	Tanimoto	3
	Canonical SMILES	1
	Graph	2
	Chiral	1
	Repetition penalty	2
Policy update	Advantage estimator	GRPO
	KL in reward/loss	Disabled
	Clip low	0.1
	Clip high	0.28
	Clip c	3.0
	Entropy coefficient	0.001
Optimization	Actor learning rate	1×10^{-6}
	Weight decay	0.01
	Gradient clip	5.0
	PPO mini-batch size	8
	PPO micro-batch size/GPU	4
	Train batch size	16 prompts
	Training length	1 epoch
Runtime	GPUs	8
	Precision/parallelism	bf16 FSDP
	Gradient checkpointing	Enabled
Trainable scope	Frozen language layer	0
	Frozen modules	Visual encoder and token embeddings
	Actor vision tower	Frozen

Additional Evaluation and Post-training Analysis

To clarify the evaluation protocol and the role of chirality, we report both the strict canonical-SMILES exact match and a chirality-ignored graph-level match. Evaluation was performed on generated SMILES strings after RDKit canonicalization (RDKit version 2025.09.1), rather than by raw string comparison or InChIKey comparison. Before canonicalization, cis/trans slash annotations were removed from both reference and prediction, and nonstandard bracketed R-group tokens were normalized to wildcard atoms following the evaluation script. The strict exact-match score then compares the canonicalized reference and prediction with tetrahedral chirality retained. The graph/no-stereo score uses the same procedure but additionally disables chirality during canonicalization. The chiral-subset score is computed only on examples whose canonical reference contains stereochemical annotations. Together, these metrics separate strict serialized-SMILES correctness, graph-level molecular identity without stereochemistry, and performance on stereochemically annotated cases. Invalid or empty predictions are counted as mismatches; valid-SMILES rates for MolSeek-OCR and MolSeek-OCR-LoRA are reported in Table S10.

As a simple uncertainty analysis, we estimated binomial sampling uncertainty from the number of images in each benchmark. Table S5 reports a conservative 95% confidence-interval half-width for a single accuracy, computed as $1.96\sqrt{0.25/N}$ and therefore maximized at 50% accuracy. Observed accuracies away from 50% have slightly smaller intervals. For a rough comparison between two independently estimated accuracies on the same benchmark, the corresponding maximum half-width for the difference is $1.96\sqrt{0.5/N}$. This approximation is intended to contextualize effect sizes rather than replace paired tests; per-image predictions are not available for all previously published baselines.

Tables S3 and S4 document the exploratory ReFT/GSPO settings, including reward components and weights, generated candidate counts, retained ReFT samples, and GSPO rollout counts. Table S6 reports the full benchmark-level chirality-aware and chirality-ignored evaluations for LoRA SFT, progressive full SFT, ReFT, and the GSPO global-step-200 checkpoint.

Table S5: Conservative binomial uncertainty by benchmark.

Benchmark	N	Single accuracy 95% half-width	Two-model difference 95% half-width
CLEF	992	3.11	4.40
CLEF-p	3555	1.64	2.32
UOB	5740	1.29	1.83
UOB-p	28580	0.58	0.82
USPTO	5719	1.30	1.83
USPTO-p	24260	0.63	0.89
ACS	331	5.39	7.62
ChemDraw	5719	1.30	1.83
Indigo	5719	1.30	1.83
staker	50000	0.44	0.62
staker-p	29228	0.57	0.81

Values are percentage points. Because ACS and CLEF are relatively small, small differences on these benchmarks should be interpreted cautiously. On the larger benchmarks, the gains from MolSeek-OCR over MolSeek-OCR-LoRA in Table S6 are substantially larger than the conservative two-model uncertainty estimate. Comparisons with previously published baselines remain limited by differences in evaluation protocol and by the lack of per-image baseline predictions for paired testing.

Table S7 summarizes the full benchmark-level pre/post post-training comparison, Table S8 reports the additional GSPO checkpoint comparison on UOB and ACS, and Table S9 provides representative examples. Overall, ReFT improved several benchmark-level exact and graph/no-stereo scores, whereas GSPO global step 200 produced only small changes relative to progressive full SFT. Neither post-training method was adopted in the final training recipe.

Case Studies

Representative cases in Table S9 illustrate typical exact-match successes and failures across supervised fine-tuning and exploratory post-training. For each fine-tuning stage, we include two examples where the base DeepSeek-OCR-2 model failed but the fine-tuned model produced a strict exact match, followed by representative failure cases from the same set of fine-tuning stages.

Figure S2 further summarizes 12 selected image-based examples in a 4-by-3 layout. These examples cover stereochemistry, complex ring or fused-ring topology, salt or R-group notation, realistic patent/catalog/journal images, perturbed-image artifacts, and a clean synthetic failure case. They supplement Table S9 with visual examples showing where au-

Table S6: Benchmark-level chirality-aware and chirality-ignored evaluation.

Method	Evaluation point	Benchmark	N	Exact match (%)	Graph/no stereo (%)	Chiral subset (%)
LoRA SFT	update 3000	CLEF	992	54.84	61.19	46.97
		CLEF-p	3555	61.97	69.25	46.81
		UOB	5740	64.27	64.32	0.00
		UOB-p	28580	43.93	44.06	0.00
		USPTO	5719	50.64	54.35	24.70
		USPTO-p	24260	56.21	59.97	32.02
		ACS	331	24.77	27.79	3.12
		ChemDraw	5719	65.01	69.61	31.63
		Indigo	5719	68.35	73.89	42.96
		staker	50000	46.29	52.24	18.60
		staker-p	29228	29.89	36.15	NA
Progressive full SFT	update 2000	CLEF	992	63.31	69.86	55.15
		CLEF-p	3555	70.66	77.89	55.82
		UOB	5740	72.61	72.70	0.00
		UOB-p	28580	49.68	49.83	0.00
		USPTO	5719	61.04	64.29	36.75
		USPTO-p	24260	65.66	69.11	44.33
		ACS	331	29.91	31.72	6.25
		ChemDraw	5719	72.13	75.45	43.82
		Indigo	5719	74.33	78.13	56.53
		staker	50000	50.50	57.01	20.97
		staker-p	29228	31.93	38.90	NA
ReFT	checkpoint 474	CLEF	992	64.21	71.37	52.73
		CLEF-p	3555	70.49	77.07	55.16
		UOB	5740	74.95	75.03	0.00
		UOB-p	28580	51.09	51.24	0.00
		USPTO	5719	61.83	65.27	36.40
		USPTO-p	24260	67.06	70.63	45.34
		ACS	331	29.61	32.33	6.25
		ChemDraw	5719	72.39	75.57	44.60
		Indigo	5719	75.90	79.59	58.17
		staker	50000	52.01	58.68	21.59
		staker-p	29228	32.66	39.68	NA
GSPO	global step 200	CLEF	992	63.10	69.86	55.15
		CLEF-p	3555	70.75	77.92	56.19
		UOB	5740	72.87	72.96	0.00
		UOB-p	28580	49.83	49.97	0.00
		USPTO	5719	61.01	64.42	36.23
		USPTO-p	24260	65.75	69.18	44.73
		ACS	331	29.61	31.72	6.25
		ChemDraw	5719	72.08	75.45	43.56
		Indigo	5719	74.37	78.13	56.35
		staker	50000	50.62	57.12	20.93
		staker-p	29228	31.95	38.95	NA

Values are percentages. Exact match compares RDKit-canonicalized SMILES with chirality retained; graph/no stereo compares the same molecules after disabling chirality during canonicalization; chiral subset reports exact-match accuracy only on references containing stereochemical annotations. NA indicates no chiral reference molecules in that benchmark subset.

Table S7: Exploratory ReFT/GSPO post-training results relative to progressive full SFT.

Method	Benchmark	N	Base exact	Post exact	Base graph/no stereo	Post graph/no stereo	Base chiral subset	Post chiral subset
ReFT	CLEF	992	63.31	64.21	69.86	71.37	55.15	52.73
	CLEF-p	3555	70.66	70.49	77.89	77.07	55.82	55.16
	UOB	5740	72.61	74.95	72.70	75.03	0.00	0.00
	UOB-p	28580	49.68	51.09	49.83	51.24	0.00	0.00
	USPTO	5719	61.04	61.83	64.29	65.27	36.75	36.40
	USPTO-p	24260	65.66	67.06	69.11	70.63	44.33	45.34
	ACS	331	29.91	29.61	31.72	32.33	6.25	6.25
	ChemDraw	5719	72.13	72.39	75.45	75.57	43.82	44.60
	Indigo	5719	74.33	75.90	78.13	79.59	56.53	58.17
	staker	50000	50.50	52.01	57.01	58.68	20.97	21.59
	staker-p	29228	31.93	32.66	38.90	39.68	NA	NA
GSPO	CLEF	992	63.31	63.10	69.86	69.86	55.15	55.15
	CLEF-p	3555	70.66	70.75	77.89	77.92	55.82	56.19
	UOB	5740	72.61	72.87	72.70	72.96	0.00	0.00
	UOB-p	28580	49.68	49.83	49.83	49.97	0.00	0.00
	USPTO	5719	61.04	61.01	64.29	64.42	36.75	36.23
	USPTO-p	24260	65.66	65.75	69.11	69.18	44.33	44.73
	ACS	331	29.91	29.61	31.72	31.72	6.25	6.25
	ChemDraw	5719	72.13	72.08	75.45	75.45	43.82	43.56
	Indigo	5719	74.33	74.37	78.13	78.13	56.53	56.35
	staker	50000	50.50	50.62	57.01	57.12	20.97	20.93
	staker-p	29228	31.93	31.95	38.90	38.95	NA	NA

Values are percentages. The base model is the progressive full-SFT checkpoint used to initialize ReFT and GSPO.

Table S8: GSPO checkpoint comparison on UOB and ACS.

Evaluation point	Benchmark	N	Exact match (%)	Graph/no stereo (%)	Chiral subset (%)
global step 100	UOB	5740	72.80	72.89	0.00
	ACS	331	29.61	31.72	6.25
global step 200	UOB	5740	72.87	72.96	0.00
	ACS	331	29.61	31.72	6.25
global step 400	UOB	5740	9.29	9.32	0.00
	ACS	331	26.28	28.10	4.69

Values are percentages. The global-step-200 checkpoint was selected for full benchmark evaluation because it was the most stable exploratory GSPO checkpoint among those inspected.

toregressive SMILES generation succeeds, where it preserves graph-level identity but misses strict stereochemical serialization, and where it fails by omitting components or changing substituent patterns.

Table S9: Representative successes and failures.

Method	Benchmark	Type	Reference SMILES	Prediction/change and note
LoRA SFT	CLEF	failure → success	CC(=O)N1CCC2=C(C1)C(C1=CC=C(C(F)(F)F)C=C1)=NN2CC(O)CN1CCC(N2C(=O)OC(C)(C)C)CC1	Malformed base output → exact match; patent image
LoRA SFT	USPTO	failure → success	[H]C(=O)N(O)CC(C)C(=O)N[C@H](C(=O)N(C)C)C(C)(C)C	Partial base output → exact match; realistic image
Progressive full SFT	CLEF	failure → success	CC(=O)N1CCC2=C(C1)C(C1=CC=C(C(F)(F)F)C=C1)=NN2CC(O)CN1CCC(N2C(=O)OC(C)(C)C)CC1	Malformed base output → exact match; patent image
Progressive full SFT	USPTO	failure → success	[H]C(=O)N(O)CC(C)C(=O)N[C@H](C(=O)N(C)C)C(C)(C)C	Partial base output → exact match; realistic image
ReFT	UOB	failure → success	CNCC1=C(C)N=C(C2=CC=CC=C2)S1	Non-SMILES base output → exact match; catalog image
ReFT	ACS	failure → success	ClC(CCC(Cl)=O)=O	Short acid chloride base output → exact match; journal image
GSPO	UOB	failure → success	CNCC1=C(C)N=C(C2=CC=CC=C2)S1	Non-SMILES base output → exact match; catalog image
GSPO	ACS	failure → success	ClC(CCC(Cl)=O)=O	Short acid chloride base output → exact match; journal image
LoRA SFT	UOB	failure	Cl.NCC1=CC=C(OC2=CC=CC=C2)C=C1	Salt component omitted
Progressive full SFT	ACS	failure	O=C(N1S[R'])CCC1=O	Fragmented aryl-sulfur output
ReFT	UOB	failure	FC(F)(F)C1=CC=C(C2=NC(CCl)=NO2)C=C1	CF_3 changed to CCF_2
GSPO	UOB	failure	FC(F)(F)C1=CC=C(C2=NC(CCl)=NO2)C=C1	CF_3 changed to CCF_2

For rows marked failure → success, the base DeepSeek-OCR-2 output failed strict exact match, whereas the listed fine-tuned model matched the reference. Rows marked failure indicate a strict mismatch by the listed model.

Table S10: Valid-SMILES rates for MolSeek-OCR and MolSeek-OCR-LoRA.

Benchmark	MolSeek-OCR-LoRA valid (%)	MolSeek-OCR valid (%)
CLEF	90.32	91.13
CLEF-p	95.11	96.65
UOB	87.67	94.70
UOB-p	91.88	95.38
USPTO	90.70	93.15
USPTO-p	91.73	94.11
ACS	78.85	79.15
ChemDraw	96.15	97.76
Indigo	97.34	97.94
staker	88.17	89.37
staker-p	91.31	92.56
All available	90.78	92.83

Validity is the percentage of predictions that could be canonicalized by RDKit after the same preprocessing used for exact-match evaluation.

Leakage Audit Analysis

As a complement to the main-text leakage audit, Table S11 reports exact-match accuracy for MolSeek-OCR-LoRA and MolSeek-OCR after removing every benchmark row whose gold structure has an exact RDKit standard InChIKey match to the cumulative two-stage training corpus. Across all available benchmark rows, 7,852 of 159,843 predictions (4.91%) were removed. On most benchmarks the kept-set accuracy differs from the original Table 2-style full-set accuracy by less than about 2 percentage points; ACS is the main exception, where removing the relatively high-overlap subset lowers accuracy by about 3.6–4.5 percentage points.

Table S11: Exact-match accuracy (%) for MolSeek-OCR-LoRA and MolSeek-OCR after removing benchmark rows whose gold structures have an exact RDKit standard InChIKey match to the cumulative two-stage training corpus.

				MolSeek-OCR-LoRA		MolSeek-OCR	
Benchmark	*N*	Overlap	Kept	Full	Kept	Full	Kept
CLEF	992	43	949	54.8	55.3	63.3	63.8
UOB	5740	284	5456	64.3	64.1	72.6	72.5
USPTO	5719	342	5377	50.6	50.9	61.0	60.9
ACS	331	48	283	24.8	21.2	29.9	25.4
Staker	50000	3206	46794	46.3	48.0	50.5	52.4
ChemDraw	5719	342	5377	65.0	64.7	72.1	71.8
Indigo	5719	342	5377	68.4	67.9	74.3	74.0
CLEF-p	3555	45	3510	62.0	62.1	70.7	70.7
UOB-p	28580	1420	27160	43.9	44.1	49.7	49.9
USPTO-p	24260	1620	22640	56.2	56.5	65.7	65.8
Staker-p	29228	160	29068	29.9	29.8	31.9	31.9
All available	159843	7852	151991	47.0	47.4	52.5	52.9

Exact-match accuracy uses the same RDKit canonical-SMILES protocol as Table 2. “Full” is the original benchmark evaluation that includes exact InChIKey overlaps; “Kept” excludes those overlapping rows. Overlap counts are based on gold structures that yield a valid InChIKey and match the cumulative training set reconstructed for the leakage audit. Aggregate accuracies are row-weighted averages over the listed benchmarks.

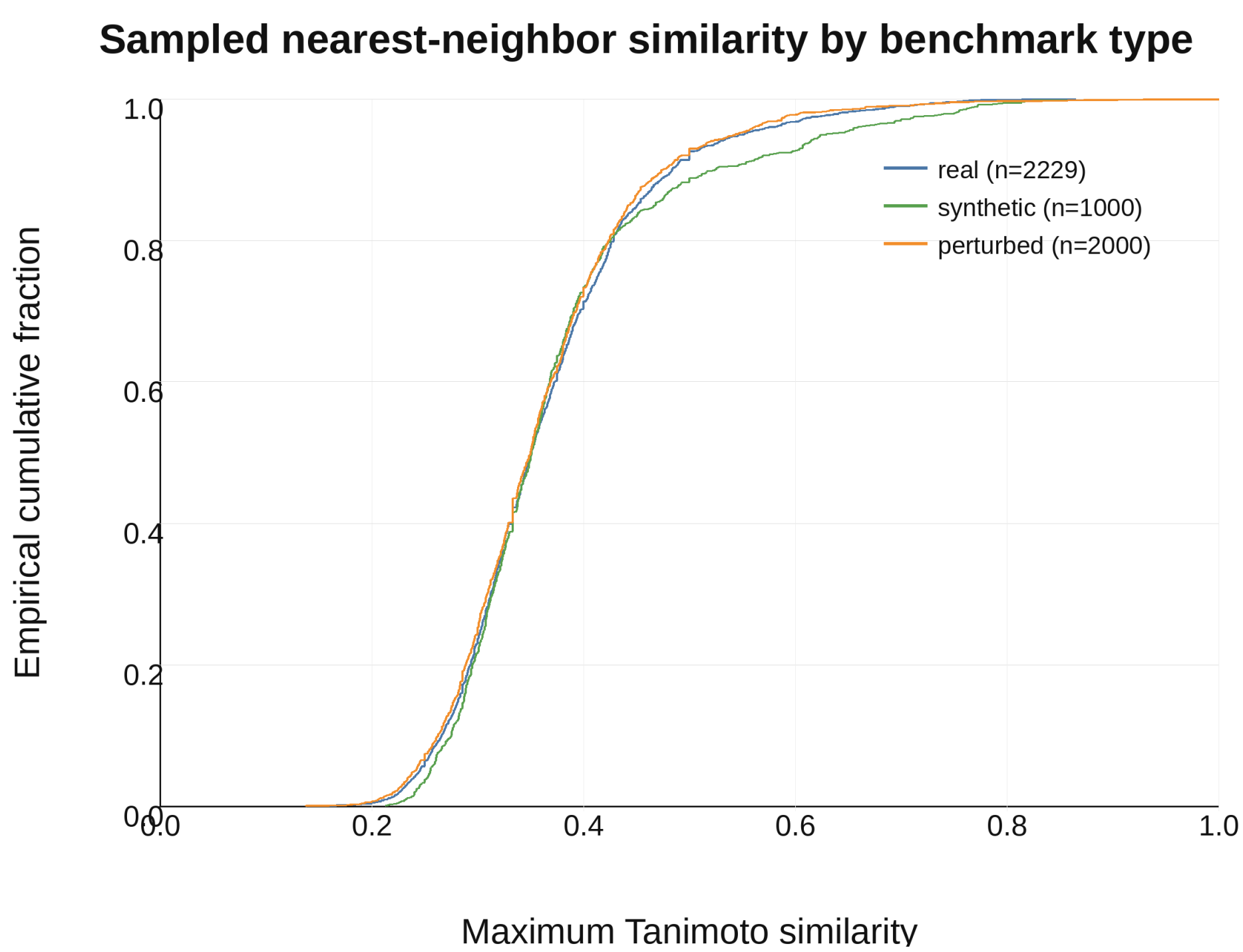

Figure S1: Empirical cumulative distribution of sampled nearest-neighbor Tanimoto similarity grouped by benchmark type after exact train–test structural overlaps were removed. Morgan fingerprints with radius 2 and 2048 bits were used, and the training side used a fixed stratified sample of 10,000 structures.

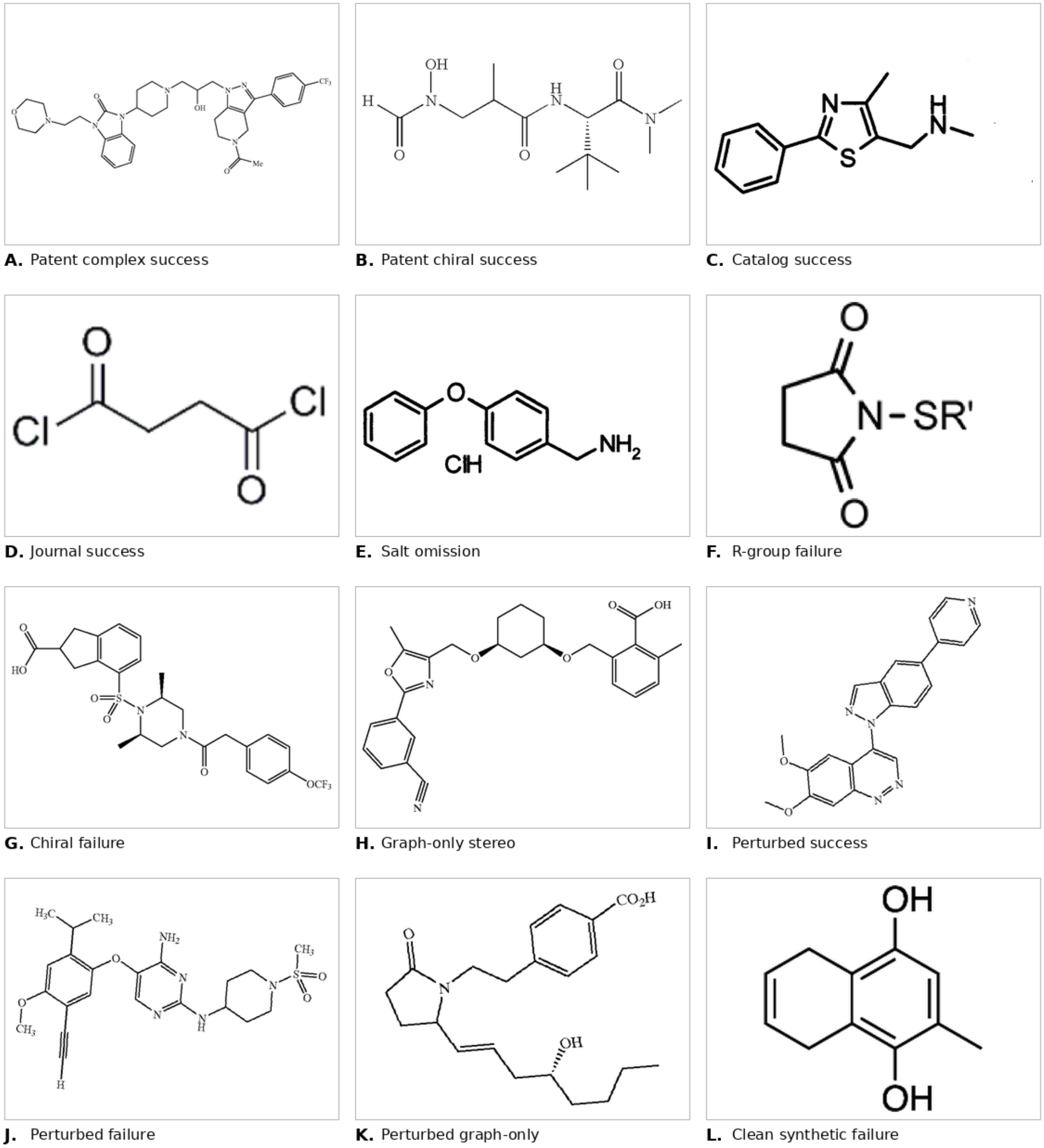

Figure S2: **Image-based case studies for representative successes and failures.** Panels A–D show cases where fine-tuning or exploratory post-training corrected malformed, incomplete, or non-SMILES base outputs across patent, chiral patent, catalog, and journal images. Panels E and F illustrate chemically meaningful failure modes involving omitted salt components and non-standard R-group notation. Panels G and H highlight stereochemistry-sensitive cases, including a strict failure and a graph/no-stereo match where the molecular graph is largely retained but stereo-sensitive exact matching fails. Panels I–K show perturbed patent examples, including one corrected case and two cases where visual distortion or stereochemical serialization still leads to mismatch. Panel L shows that errors can also occur for clean synthetic ChemDraw-style images, indicating that some failures reflect topology or unsaturation parsing rather than image noise alone.
